# Pre- and Post-Procedural Considerations and Thread Types for Thread Lifting

**DOI:** 10.3390/life15010085

**Published:** 2025-01-12

**Authors:** Gi-Woong Hong, Jovian Wan, Song-Eun Yoon, Sky Wong, Kyu-Ho Yi

**Affiliations:** 1 Samskin Plastic Surgery Clinic, Seoul 06577, Republic of Korea; 2 Medical Research Inc., Wonju, Republic of Korea; 3 Brandnew Aesthetic Surgery Clinic, Seoul, Republic of Korea; 4 Leciel Medical Centre, Hong Kong; 5 Division in Anatomy and Developmental Biology, Department of Oral Biology, Human Identification Research Institute, BK21 FOUR Project, Yonsei University College of Dentistry, 50-1 Yonsei-ro, Seodaemun-gu, Seoul 03722, Republic of Korea; 6 Maylin Clinic (Apgujeong), Seoul, Republic of Korea

**Keywords:** rhytidoplasty, facial muscles, superficial musculoaponeurotic system, rejuvenation, surgical procedures, minimally invasive

## Abstract

Facial thread lifting has emerged as a minimally invasive alternative to traditional face-lifting procedures, with particular emphasis on U-shaped and I-shaped barbed threads. This review analyzes the anatomical considerations, procedural techniques, and clinical outcomes of different thread types for facial rejuvenation. The study examines the mechanical principles and lifting mechanisms of U-shaped “suspension type” threads versus I-shaped threads, highlighting their distinct characteristics and applications. The results indicate that U-shaped threads provide strong lifting effects, with success rates reported at 85–90% in achieving visible tissue elevation when anchored in the temporal area. However, these threads carry higher risks of complications, including bleeding (15–20%), dimpling (12–18%), and tissue damage at exit points (5–10%). In contrast, I-shaped threads demonstrate advantages in minimizing tissue trauma and patient discomfort, with complication rates below 5%, though they may provide less dramatic lifting effects. The study concludes that optimal outcomes are achieved through careful patient selection and customized combination approaches rather than reliance on a single thread type. Future directions point toward the development of hybrid techniques that combine the strengths of both thread types to maximize efficacy while minimizing complications.

## 1. Introduction

The evolution of facial aesthetics has led to an increasing demand for minimally invasive procedures that effectively address age-related changes while minimizing downtime and complications. Thread lifting has emerged as a significant advancement in this field, offering a middle ground between non-invasive treatments and traditional surgical interventions. This technique employs specialized sutures, often barbed, to mechanically reposition descended facial tissues, creating a lifting effect that enhances facial contours and addresses signs of aging.

A critical element in understanding thread lifting is the anatomy of the face, particularly the Superficial Musculo-Aponeurotic System (SMAS). The SMAS is a fibrous layer of tissue that lies between the skin and deeper facial structures, playing a key role in facial expressions and skin attachment. It acts as a foundation for many lifting procedures, as repositioning the SMAS layer can result in more durable and natural-looking outcomes. A thorough understanding of the SMAS’s varying thickness and mobility across different facial regions is essential for achieving optimal thread lifting results while minimizing complications [1,2,3].

Despite the growing popularity of thread lifting, there remains a significant knowledge gap regarding the comparative efficacy, safety, and long-term outcomes of different thread types, specifically U-shaped and I-shaped barbed threads. These threads differ not only in their design but also in their mechanical principles and applications. U-shaped threads are commonly anchored in the temporal area and provide robust lifting effects, but they are associated with higher risks of complications, such as dimpling and tissue damage at exit points. In contrast, I-shaped threads are designed to minimize tissue trauma and discomfort but may offer subtler results [4,5].

This review aims to bridge this gap by analyzing the anatomical considerations, procedural techniques, and clinical outcomes of U-shaped and I-shaped threads. By synthesizing existing evidence and addressing critical issues such as complication rates, patient selection, and hybrid approaches, this study provides practitioners with an evidence-based framework for optimizing thread lifting procedures. Furthermore, it highlights the potential for hybrid techniques to overcome the limitations of individual thread types, paving the way for more effective and personalized treatments [5,6].

## 2. Material and Methods

A systematic literature search was conducted to analyze anatomical considerations, procedural techniques, and clinical outcomes associated with thread lifting, focusing on U-shaped and I-shaped barbed threads. Databases including PubMed, MEDLINE, and Ovid were searched using predefined keywords such as “thread lifting”, “facial rejuvenation”, “barbed threads”, “U-shaped threads”, “I-shaped threads”, “SMAS anatomy,” and “minimally invasive lifting”. The search spanned studies published up to October 2024.

Inclusion criteria were as follows: studies addressing the use of barbed threads for facial rejuvenation; articles with documented outcomes related to U-shaped or I-shaped thread techniques; reports that included anatomical analyses, procedural descriptions, or complication rates; studies in English with human participants.

Exclusion criteria were as follows: studies focusing solely on non-barbed threads or threads used for non-facial areas; case reports, opinion pieces, or studies lacking outcome data.

The PRISMA (Preferred Reporting Items for Systematic Reviews and Meta-Analyses) guidelines were followed to ensure methodological rigor and transparency. Of the 48 articles initially identified, 37 were reviewed as full texts, and 15 studies were included in the final synthesis. Data extraction focused on thread design, procedural techniques, complication rates, and comparative efficacy.

Figures and tables in the manuscript were created by the authors based on the synthesized data and clinical observations.

## 3. Result

*Pre- and Post-Procedural Considerations*

**Figure 1:**
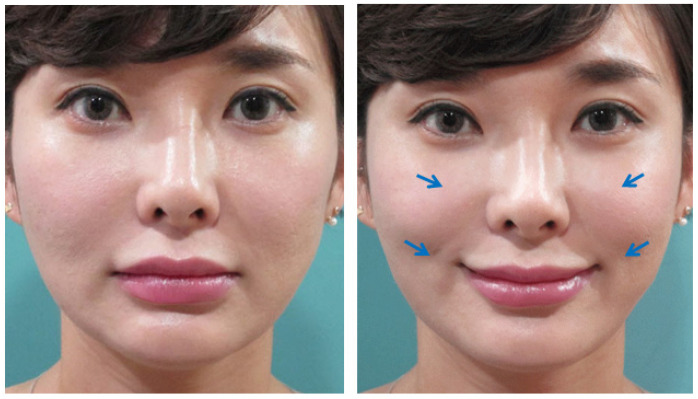
Soft tissue change of the face when smiling. When smiling, the soft tissues of the face undergo distinct movements: the anterior malar area shifts in an anterosuperior direction, while the paranasal and perioral areas move laterosuperiorly (blue arrows). These movements result in notable proportion changes between the mid and lower face, characterized by increased volume in the midface region and a reduction in lower face volume, creating a slimmer appearance of the lower face. Created by the authors.

Our findings indicate that improved facial contours can be achieved through barbed thread lifting, with the most natural and ideal form resembling the facial shape created during a smile. In comparison to a neutral, expressionless state, smiling causes the skin and soft tissues of the mid and lower face to be pulled upwards due to the contraction of the upper lip elevator muscles. Upon measurement, it is observed that the zygomatic area tissues are pulled anteriorly and superiorly, while the perioral tissues are pulled laterally and superiorly. Consequently, when we smile, the lower face narrows and becomes more defined, with sagging tissues lifted upwards and the anterior zygomatic area becoming more prominent. By utilizing barbed thread lifting to elevate sagging areas and recreate this overall facial shape, it is possible to achieve both rejuvenation and a slimmer, more aesthetically pleasing facial contour (Figure 1) [7].

As previously mentioned, to maximize the effects of barbed thread lifting, it is often necessary to insert thick barbed threads deeper than the superficial subcutaneous tissue layer. To appropriately lift the SMAS layer, which is located deeper than the superficial fat compartment, it is crucial to understand that the thickness of the SMAS layer varies across different facial regions. Typically, the SMAS layer is thicker anterior to the ear, visible to the naked eye as a white, tough membrane covering the deep fat layer. As it progresses anteriorly across the face, the SMAS layer thins, becoming nearly transparent near the nasolabial folds, giving the appearance of absence. However, it should be noted that the SMAS layer extends thinly even medial to the nasolabial folds. By adjusting the force applied when moving the cannula according to the thickness of the SMAS layer in different facial areas, one can prevent overly deep penetration that might damage facial muscles or cause the thread to perforate into the oral cavity (Table 1) [8,9].

**Figure 2:**
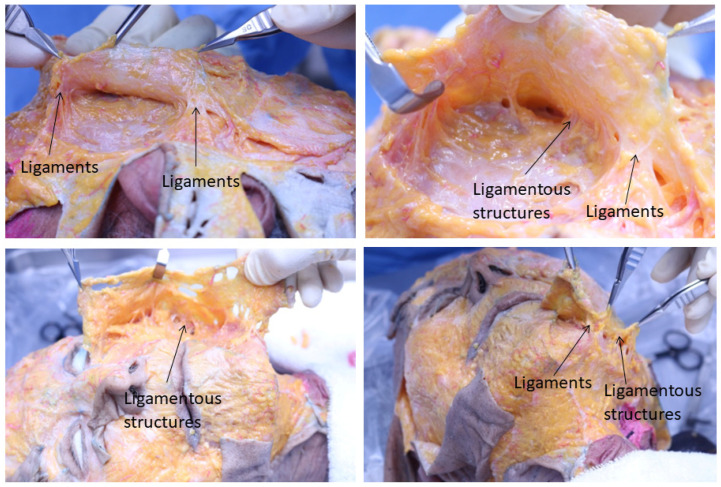
Original ligaments and ligamentous structures on entire face. This figure illustrates the comprehensive layout of facial ligaments and ligamentous structures across the entire face, showing their natural anatomical arrangement. Created by the authors.

The facial space, including the deep fat layer beneath the SMAS, is a relatively mobile area. Using the pinch technique, where the patient’s facial skin and soft tissues are grasped and lifted with the fingers and one can feel the smooth gliding and stretching of the tissues above this space. The facial space is compartmentalized by tight original facial ligaments and less rigid ligamentous structures, primarily consisting of fat and soft tissues. When lifting sagging facial areas upward using barbed threads, the facial space and the relaxed ligamentous structures move readily. However, the very rigid original facial ligaments do not stretch easily, and the resistance of the skin attached to these ligaments may cause dimpling. Caution is necessary in these areas, and adjustments in depth or direction of thread placement may be required. Therefore, a thorough understanding of the anatomical distribution of mobile facial spaces, relaxed ligamentous structures, and firmly anchored original retaining ligaments before barbed thread lifting can lead to more efficient lifting procedures (Figure 2).

After comprehending these anatomical differences, it is beneficial to determine the appropriate vectors for lifting based on the target skin and soft tissues (Table 2).

The depth and direction of thread insertion should vary across facial regions to achieve a three-dimensional lifting effect rather than a simple linear pull. When designing the entry points and vectors for thread insertion, several factors should be considered, including the elasticity and weight of the skin and soft tissues to be lifted, the location of rigid original retaining ligaments and age-related lax ligamentous structures, the degree of facial volume loss and consequent skin sagging in different areas, the thickness and toughness of the SMAS, and the position and direction of important facial muscles that affect facial expressions. Considering these elements in the design and execution of the procedure can minimize side effects while maximizing natural-looking and effective results.

Lastly, regarding the mechanism of action of barbed threads, previous vertical lifting techniques for the lateral face involved pulling the distal part of the thread through the skin, similar to long U-shaped barbed threads, believing this allowed for maximum tissue engagement. However, in practice, to prevent dimpling at the exit point, a portion of the externalized thread, including some of the internal thread, must be pulled and cut. Consequently, the actual tissue-gripping portion of the barbed thread is not significantly different from methods where the thread end remains buried beneath the skin. Therefore, I-type barbed threads that do not penetrate the skin externally are now more commonly used for lifting. Another reason for this shift is that to externalize the distal part of thick threads, the cannula tip must be somewhat sharp. In patients with very thin skin and soft tissues, this sharp tip can cause various injuries to important structures located close to the subcutaneous layer, even when using a cannula. By eliminating the need to penetrate the skin from the outset, a blunt-tipped cannula can be used throughout the procedure, allowing for safe barbed thread lifting in the deep layers of the lateral face, even in patients with thin and lean skin [10,11].

Prior to delving into the specific methods of barbed thread lifting for different facial areas, it is essential to consider the overall aspects of the procedure, including patient consultation, treatment planning, and post-procedural precautions.

Typically, when discussing facial changes associated with aging, many people consider the deepening of expression-induced wrinkles, caused by thinning connective tissue and loss of elastic fibers within the skin, as the most significant sign of aging. However, as elucidated by Australian plastic surgeon Bryan Mendelson in his book “In Your Face”, the most crucial sign of aging is the change in facial shape.

The desire to appear younger than one’s actual age is a universal aspiration, transcending age, gender, ethnicity, geographical location, and social status. As we age, the supporting structures that maintain facial expressions by firmly anchoring the skin and tissues gradually become lax. This occurs due to the reduction in the volume of deep fat layers beneath the SMAS. Despite the increased importance of these supporting structures as skin and soft tissue elasticity diminishes, they fail to provide adequate support. Consequently, the skin and soft tissues of the face become increasingly saggy.

As these sagging tissues accumulate along the jawline, the face appears to widen and lengthen progressively. This transformation of facial shape, which deviates from the standards of a youthful appearance, is considered the most significant sign of aging.

Among minimally invasive procedures, barbed thread lifting is considered the most effective method to slow and improve these facial shape changes. Performing thread lifting in the early stages of aging can maximize its effects and naturally decelerate the aging process. Recently, thread lifting has become popular not only for age-related facial changes but also among younger Asian patients who dislike wide faces and those seeking to improve skin and soft tissue sagging after facial contouring surgery or liposuction [10,11,12].

Barbed thread lifting can be beneficial for various patient groups and conditions. It is suitable for patients experiencing common aging symptoms such as loss of skin elasticity, wrinkle formation, and facial widening due to sagging skin and soft tissues. It can also benefit those who want a tighter and slimmer facial appearance but have found limitations with common skincare, botulinum neurotoxin, or device treatments.

For younger individuals seeking a slimmer face without bone surgery, where reducing soft tissue volume alone is insufficient, thread lifting can be an effective option. It can also improve soft tissue sagging in patients who have undergone facial contouring surgery involving bone cutting or reduction, where the supporting structures have been altered.

Thread lifting is also useful for patients wanting to correct skin laxity and sagging after facial liposuction, where the skin has lost elasticity and appears deflated. Conversely, it can address issues of downward migration of transplanted fat in patients who have undergone facial fat grafting procedures to add volume.

Furthermore, thread lifting can improve areas that are difficult to treat with fillers or toxins alone, such as eyebrow ptosis, nasolabial folds, marionette lines, and double chin correction. These applications make thread lifting a versatile option for addressing various facial aesthetic concerns across different age groups and previous treatment histories.

Additionally, in the past, face-lifting surgery was recommended when excess skin of 2 cm or more was observed when pulling the skin towards the ear. However, with recent improvements in thread quality and the development of monofilaments capable of producing volume effects with excellent skin regeneration properties, the limitations of thread lifting compared to surgery are being overcome. Increasingly, patients are opting for thread lifting based on their preferences after being informed of its limitations compared to surgery.

Furthermore, thread lifting is emerging as a good alternative for patients who have previously undergone face-lifting surgery but are experiencing skin and soft tissue sagging again over time and are reluctant to undergo another major surgery. Considering these trends, it appears that the role of thread lifting in aesthetic medicine will continue to grow, much like how fillers have evolved from their initial applications to now serving as alternatives to fat grafting as their quality improved and diverse types became available over time.

During pre-procedure patient consultation, it is crucial to listen to the patient’s concerns about their face, analyze their current condition, and explain not only the effects of barbed thread lifting but also the procedure process and potential post-procedure symptoms and side effects. It is also important to clearly explain the inevitable limitations of the procedure if it seems unlikely to fully satisfy the patient’s requirements, and to prevent unrealistic expectations.

Before the procedure, carefully examine the patient’s condition and plan how to perform the procedure to meet their requirements and achieve the best results. Take photographs before designing the procedure. Having pre-procedure photos allows for accurate diagnosis by comparing before and after appearances if problems arise or the patient expresses dissatisfaction. This enables more objective explanations of post-procedure progress, helps in deciding on additional procedures, and aids in persuading patients if their perceptions are incorrect. Printing and referencing pre-procedure photos during the procedure can lead to more accurate treatment and prevent mistakes such as confusing areas or switching left and right sides.

Initial swelling and bruising may occur, potentially lasting 2–4 weeks if large blood vessels are affected. Patients taking blood circulation improvers like aspirin or menstruating women are prone to bleeding, so it is best to consider this when scheduling the procedure. If surgery is unavoidable in these cases, explain the increased likelihood of bruising and prescribe vitamin K ointment to minimize bruising.

After the procedure, it is advisable to keep the head elevated above the heart to prevent swelling from concentrating in the facial area. For about a week post-procedure, patients should be instructed to sleep on their back to avoid pressure on the treated areas. Patients should be informed that they may experience pain when moving their mouth while speaking or making expressions, especially when eating or laughing widely. They may also feel tightness, itching, and warmth for a few days. Prescribe antibiotics, anti-inflammatory drugs, and pain relievers as needed.

If thick barbed threads are unintentionally inserted too deeply into the fat layer below the SMAS, patients may have difficulty opening their mouth slightly and may experience a tingling sensation even at rest. These sensory abnormalities may persist for up to a month in severe cases, so caution is always necessary.

For severe bruising or swelling, recommend cold compresses, which can also help with sensitive red skin, pain, and sensory abnormalities. Inform patients that they can wash their face or take a light shower on the evening of the procedure day, and that makeup application and light exercise are possible from the day after the procedure. Advise avoiding saunas, spas, swimming pools, and alcohol consumption for about a week after the procedure. For 1–2 weeks post-procedure, depending on the patient’s condition, it is best to avoid opening the mouth wide, eating hard foods, and any facial massages or strong pressure on the face.

Instruct patients to return for a follow-up visit within a day or two after the procedure to check for any abnormalities and to address any symptoms that may appear as swelling subsides. The most common complaint is skin dimpling or irregularities where the threads are inserted, as the barbs may grip the skin and soft tissue too tightly. While these issues often improve gradually with normal facial movements and daily activities, they should be addressed promptly to prevent fibrosis between the thread and tissue, which can lead to persistent depressions.

If massaging or molding the skin surface does not resolve the irregularities, injecting saline solution into depressed areas before massaging or molding can help release the skin caught by the barbs more easily. Although most severe symptoms improve slowly over 1–2 months, addressing these issues immediately after the procedure can reduce patient anxiety and complaints during the recovery period [13].

Even after these post-procedural treatments, some skin irregularities may remain visible at the thread boundaries, and linear depressions may appear along the thread silhouette. The irregularities at the thread boundaries are often due to structural changes in the skin where threads are inserted versus areas without threads, rather than the skin being caught in the barbs themselves. This is particularly noticeable in skin that has lost elasticity. Linear depressions along the thread contour can occur if the thread is positioned too close to the skin surface or if the skin and soft tissue are thin and soft. In such cases, if a smoother skin appearance is desired, injecting a soft consistency filler along the depressed areas can help create a more even and aesthetically pleasing skin surface.

As time passes, swelling subsides, and pain decreases, allowing for more natural facial movements. With increased movement of facial muscles and skin, there is a possibility that the thread contours may become visible or palpable, and the thread ends may potentially protrude through the skin or into the oral cavity. It is important to inform patients about these possibilities in advance. This preemptive communication allows for better management of patient expectations and facilitates smoother treatment if such issues do occur, without damaging the patient-practitioner relationship.

These considerations highlight the importance of thorough patient education, careful technique, and post-procedural care in thread lifting procedures. They also underscore the need for practitioners to be prepared for potential complications and to have strategies in place for addressing them promptly and effectively.

## 4. Clinical Differences Between U-Shaped and I-Shaped Barbed Thread Lifting

This section aims to summarize the actual lifting mechanisms and most efficient methods when using relatively thick barbed threads for facial contouring. The focus is on the two most clinically prevalent types: the long U-shaped threads that are bent in the middle, and the relatively shorter, straight I-shaped threads.

Clinically, many physicians classify long U-shaped threads and short I-shaped threads as fixed (or anchoring) threads and non-fixed (or floating) threads, respectively, based on their principle rather than their shape or form. However, the author believes this classification does not align with the basic principles of the threads.

**Figure 3:**
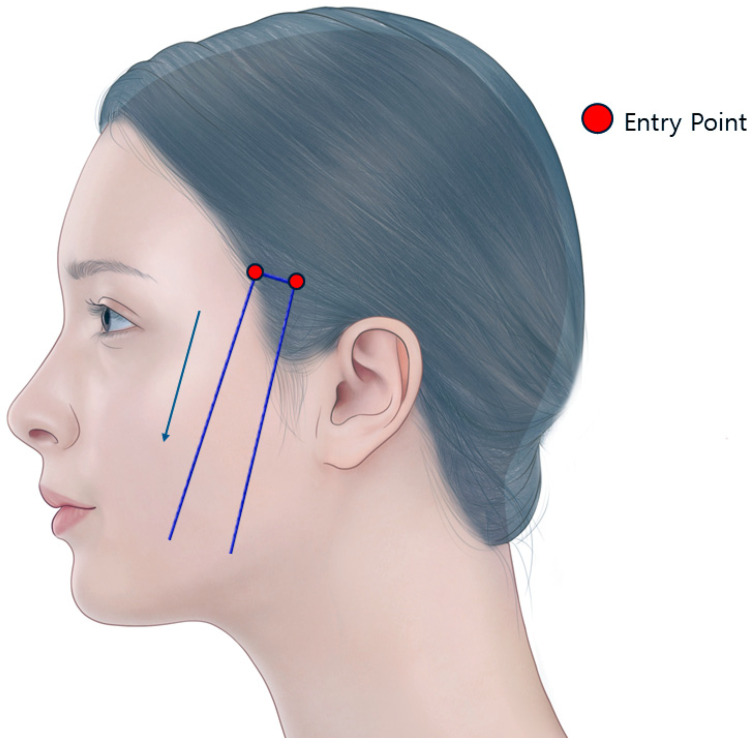
This illustration shows the typical placement of U-shaped barbed threads used in facial lifting procedures, with the blue arrows indicating the direction and positioning of the threads. Created by the authors.

U-shaped threads are longer than two overlapping I-shaped threads. When lifting the perioral and cheek areas, the middle portion without barbs is typically inserted into the temporal area. The barbed portions on both sides are then inserted from the temporal area towards the lower face. This method pulls up the skin and soft tissues of the treatment area and is commonly referred to as fixed thread lifting (Figure 3).

**Figure 4:**
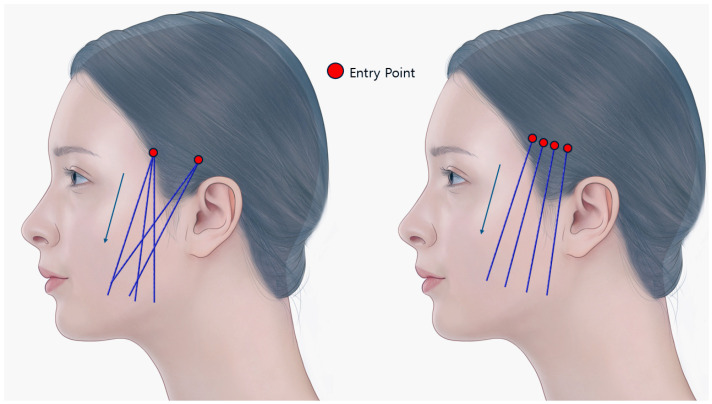
Usual design of I-shape barbed thread lifting. This figure shows the typical arrangement for I-shaped barbed thread placement in facial lifting procedures. The thread that is used are 10 cm length of Secret Illusion (Hyundaimeditech Inc., Wonju, Republic of Korea) and Sihler thread (Sihler Inc., Seoul, Republic of Korea). The arrows indicate the placement of the threads. Created by the authors.

I-shaped thread lifting, often called non-fixed thread lifting, does not involve anchoring the thread in the temporal area. Instead, the entry point is usually set inside or near the side hairline, and the thread is inserted straight. Early models were essentially shorter versions of U-shaped threads with a single bidirectional type, gripping tissue from both ends towards the center. However, modern I-shaped lifting threads, even if bidirectional, have adjusted barb shapes and numbers. The barbs towards the head serve only to prevent the thread from sliding towards the lower face by catching on firm upper tissues. Meanwhile, the downward-facing barbs grip and pull up the loose tissues of the mid and lower face (Figure 4). The thread that is used are 10 cm length of Secret Illusion (Hyundaimeditech Inc., Wonju, Korea) and Sihler thread (Sihler Inc., Seoul, Korea).

This detailed explanation provides a comprehensive understanding of the structural and functional differences between U-shaped and I-shaped threads, highlighting their respective mechanisms of action in facial contouring procedures.

Recent trends in I-shaped barbed threads have advanced a step further. Rather than forcefully pulling sagging tissue to its maximum extent, the focus is now on how efficiently the relocated tissues can be maintained in place. This is achieved by slightly lifting the chin in a supine position, naturally moving the loose and sagging lower facial tissues towards the firmer tissues near the head. The success of the procedure largely depends on how effectively these moved tissues can be kept in their new position [14].

To understand why the term “fixed method” is used for long U-shaped threads, let us examine the procedure design for lifting the lower face in more detail. Typically, the thread is inserted into the temporal area, with the non-barbed middle portion anchored to the deep temporal fascia or at least the innominate fascia, the firmest tissue layers in the temporal region. The barbed portions on both sides are then inserted downwards towards the cheeks, passing through the skin. When the desired area is reached, the thread is pulled out through the skin, lifting the skin and tissues in this area.

The barbs on both sides of the thread, centered around the non-barbed portion, are oriented outwards. When inserted parallel from the head to the lower face in a U-shape, both threads have their barbs oriented in the same direction: the forward direction of the barbs faces the lower face, while the reverse direction faces the temporal area. This arrangement allows both threads to pull the lower tissues upwards, effectively using one long thread to perform the function of two.

Regardless of the design used, when the ends of both barbed threads are pulled out through exit points in the skin and then tugged, the barbs on either side face opposite directions. Pulling the left thread applies force in the reverse direction of the right thread’s barbs, and vice versa. This creates a gripping and pulling action on the tissues.

As both barbed threads progress inside the skin, their barbs act in the forward direction. When the threads that have passed through the tissue are pulled out through the skin, the force acts in the reverse direction of the barbs on the opposite thread. This creates a balance between forward and reverse forces, naturally moving loose tissue towards the firmer areas near the head.

When using a U-shaped design for lifting the lower face, a unique problem arises. Unlike other designs, both sides of the thread have their forward direction pointing from the upper part towards the lower face, similar to using two unidirectional-type barbed threads. This requires the non-barbed upper portion to be firmly anchored in strong tissue to keep the two strands separated. If this part fails to anchor properly in firm tissue, it cannot withstand the pulling forces from both barbed sides, resulting in a “cheese-wiring effect” where the threads slip downwards in the direction of the barbs.

However, it is important to note that the principle behind the thread not moving or falling downwards while continuously pulling the tissue is not primarily due to the non-barbed portion being fixed in the deep, firm temporal fascia. Rather, it is because of the balance created between the forward and reverse directions of the barbs on both sides of the thread, as explained earlier.

In other words, while the barbs on both threads are oriented to pull the loose tissues of the lower face towards the firm temporal fascia, the tendency of the thread to unravel and slip downwards in the forward direction is counteracted by the reverse action of the barbs on the opposite thread [15,16].

Therefore, when using U-shaped threads, there are two main reasons why the thread might unravel and slip downwards. The barbs on both sides of the thread should create a balance of forces in forward and reverse directions. If the pulling force of one thread weakens, it cannot withstand the downward force of the other thread, causing the thread to move. Additionally, if the non-barbed portion of the U-shaped thread is not properly anchored in firm tissue, issues can arise. The two strands need to be separated for the forward direction of one thread’s barbs to balance with the reverse direction of the other barb. If the tissue in the middle non-barbed portion is weak and soft, it fails to keep the two strands apart. When the two strands are not adequately separated, unlike I, L, or V designs, in a U-shaped design, both strands point in the same direction. This eliminates the counterbalancing function, making them act like two separate unidirectional barbed threads, with no force to prevent downward movement.

**Figure 5:**
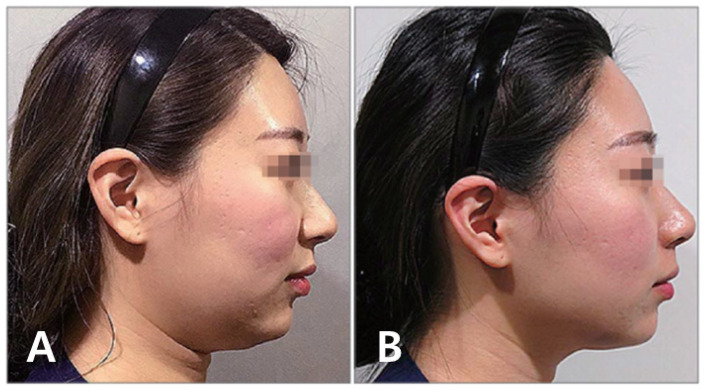
Double chin and jaw line barbed thread lifting—pre- (**A**) and post-procedure (**B**) photos. This figure presents comparative photographs showing the results of barbed thread lifting for double chin and jawline enhancement, displaying both before and after treatment images. Created by the authors.

The fact that the non-barbed middle portion is not actually fixing and pulling the tissue becomes evident when using long U-shaped threads in other designs. For instance, when inserting a U-shaped thread in an I-shape design to correct a double chin, the loose, bulging soft tissue between the lower jaw and neck (where the non-barbed portion passes) gets pulled towards the firm mastoid fascia surrounding the mastoid bone below the ear (where the barbed ends pass). This is opposite to when the thread is anchored in the temporal area. The firmer tissue at the ends of the barbed threads allows the loose, bulging area of the double chin in the middle (where the non-barbed portion is located) to be pulled towards the firm ends, flattening and improving its shape when the thread is tugged (Figure 5).

In defining terminology, a “fixed” method should refer to a technique where the sagged area is pulled up and secured to a firmer tissue area to prevent it from falling back down, even when pulled downwards. However, in U-shaped barbed thread lifting of the lower face, passing the non-barbed middle portion through firm tissues like the deep temporal fascia or innominate fascia is not for fixation. Rather, it is to withstand the load when the barbed portions grip and pull the loose, sagging tissue below. If this non-barbed portion were to catch on thin, weak tissue, it could not withstand the tension from the barbed parts and would tear, preventing the barbs on both sides from acting in opposite directions and losing their counterbalancing effect. In practice, after hooking the thread onto the firm temporal fascia but before inserting the barbed portions, the non-barbed section can be seen moving and sliding freely from side to side.

In conclusion, U-shaped threads are essentially elongated versions of the initial I-shaped bidirectional barbed threads. After inserting both barbed ends into the tissue, the thread appears fixed not because the non-barbed temporal portion is actually anchored to the fascia, but because, as explained earlier, the oppositely oriented barbs on the right and left sides of the thread pull the tissue while counteracting each other’s forward movement, creating a balance that makes the thread appear fixed at the temporal area.

Therefore, the author argues that a truly “fixed” method, regardless of the thread type used, involves creating a fixation point proximally by tying the thread or attaching it to a fixing device like a mesh to prevent downward movement. Simply passing the non-barbed portion of a long U-shaped thread through temporal tissue does not constitute a fixed method.

From a clinical perspective, the author suggests that thick barbed threads for lifting can be broadly categorized into two types: long U-shaped threads and I-shaped threads. While both share the same basic principle and can be used in various procedural designs, they are named according to their most common usage. I-shaped threads come in various lengths, thicknesses, and barb shapes but are fundamentally designed for I-shaped applications only. If one were to give another name to the long U-shaped thread for ease of reference, it could be called a “suspension-type” lifting thread, reflecting how the non-barbed middle portion is suspended over the temporal area.

The next important consideration with U-shaped barbed threads is that for the barbs on both sides to work in opposite directions, the ends of the threads must pierce through the skin. While a guide cannula can minimize tissue damage during insertion, there is still a risk of bleeding or structural damage when the thread exits the skin. Skin dimpling or irregularities at the exit points also require attention.

As mentioned earlier, many U-shaped barbed threads now come with long needles attached to the ends, allowing for use without a guide cannula, especially for thinner threads around 23 G. These threads operate on the same principle as Silhouette Soft, just replacing cones with barbs. However, even though the needle tips are designed to minimize tissue damage, they must still be somewhat sharp to pass through tissue, unlike blunt-tipped cannulas. This increases the risk of damaging blood vessels, nerves, and other structures compared to cannulas.

**Figure 6:**
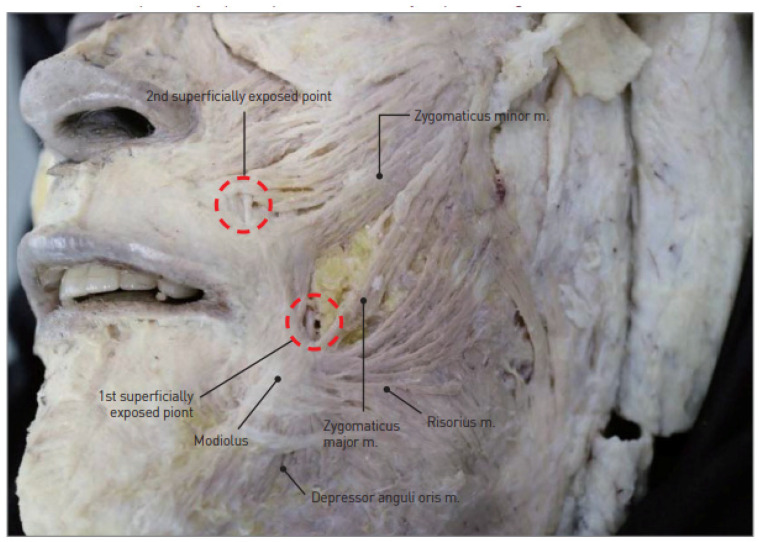
Two superficially exposed points of facial artery in perioral region. This illustration identifies the two points where the facial artery becomes superficially exposed in the perioral area. Created by the authors.

The author recounts a personal experience where using such a long-needled U-shaped thread near the mouth area resulted in hitting a superficial facial artery, causing persistent bleeding, severe bruising, and swelling. Such complications can take a long time to resolve and negatively impact the procedure results and duration (Figure 6).

Moreover, many women today have very thin skin with little soft tissue and fat. Carelessly moving the needle can easily damage nerves, parotid ducts, or glands that are closer to the skin surface than expected. This risk is particularly high in thin patients and requires extra caution.

Considering these factors, it is advisable to choose between U-shaped and I-shaped threads based on the patient’s condition, desires, and characteristics when performing thread lifting procedures.

When selecting threads based on the degree of skin and tissue sagging, it is challenging to establish clear criteria for which thread suits which level of sagging. This is because the appropriate choice can vary depending on both the patient’s preferences and the degree of lifting the physician aims to achieve. Some thread companies showcase dramatic before-and-after photos of ideal cases in their promotions. To instill more confidence, it would be helpful if these companies provided detailed explanations of their products’ indications, including both favorable and limiting cases. This would allow for more appropriate thread and procedure selection based on individual patient cases.

Once personal criteria are established, thread selection should be based on the degree of skin and tissue sagging. For minimal sagging that does not require strong force, lighter threads with less foreign body sensation or discomfort can be used. However, for severe sagging requiring stronger tensile strength and stress, appropriate products should be chosen. If not using fixed methods like tying at the top or anchoring to a mesh, it is advisable to use long U-shaped threads with good pulling strength or I-shaped threads with strong pulling power (considering thread thickness, barb tensile strength, stress, etc.), or adjust the number of threads used. However, no single thread type is universally superior. For instance, while U-shaped threads in the temporal area provide good lifting effects, the level of discomfort for the patient and potential complications like bleeding, skin dimpling, or irregularities at the exit points should also be considered.

The ideal product minimizes patient discomfort and side effects while providing adequate results. To achieve this, the current trend is to combine different types of threads with varying mechanisms of action, rather than using just one type.

Excessive pulling can lead to an unnatural appearance lasting 1–2 months or longer, with skin irregularities persisting even longer. Some practitioners suggest maximum initial pulling followed by massage to adjust the lifting force. However, this approach can lead to either loss of effect due to over-massage or persistent tightness causing severe patient complaints, contradicting the minimally invasive nature of the procedure.

Therefore, given the pros and cons of different thread shapes, it is crucial to understand the characteristics of each thread type and the quality offered by different companies. The practitioner should choose threads that align with the patient’s condition and their own treatment philosophy [17,18].

Hybrid thread lifting techniques combine the strengths of U-shaped and I-shaped threads, effectively addressing the limitations of single-thread approaches. By utilizing U-shaped threads for anchoring and robust lifting in areas like the lower face and jawline, while applying I-shaped threads to refine midface contours and treat delicate regions such as the nasolabial folds or perioral areas, this method achieves comprehensive facial rejuvenation with minimized tissue trauma.

The combination of different thread types also enhances the longevity of results. Hybrid techniques distribute tension more evenly across tissues, reducing the risk of thread migration or loosening. This balanced approach leads to lifting effects that can last up to 18–24 months, offering a significant advantage over single-thread approaches.

Additionally, hybrid methods reduce complications by leveraging less invasive I-shaped threads in regions prone to risks, such as areas with thin skin or high vascularity. This targeted application minimizes adverse effects like dimpling, bruising, or sensory abnormalities, ensuring safer outcomes for patients.

Hybrid techniques also allow for customized aesthetic results, tailored to meet individual patient goals. This flexibility makes it possible to achieve dramatic lifting in the jawline with U-shaped threads while creating natural, refined improvements in the midface using I-shaped threads. The combination delivers a balanced and harmonious rejuvenation, offering superior results that address both functional and aesthetic concerns.

This approach emphasizes the importance of personalized treatment plans, considering not just the technical aspects of the threads but also patient comfort and long-term satisfaction. It also highlights the evolving nature of thread lift procedures, with combination techniques becoming more prevalent to optimize results while minimizing side effects.

## 5. Discussion

Among minimally invasive techniques, thread lifting is unique in its ability to mechanically lift sagging tissues. This sets it apart from other modalities like fillers and laser treatments, which primarily address volume loss or skin quality but do not physically reposition facial structures.

Recent advancements in thread design, particularly the development of =PDO threads, have significantly enhanced the safety, efficacy, and versatility of thread lifting procedures. PDO threads, composed of a biodegradable polymer that naturally dissolves within the body over several months, offer a unique combination of mechanical lifting and collagen-stimulating properties, leading to improved patient outcomes.

One major advancement in PDO thread design is the introduction of multidirectional barbs. These barbs increase the thread’s grip on soft tissues, providing a more secure and effective lift. Unlike earlier designs with unidirectional or bidirectional barbs, multidirectional barbs distribute tension more evenly, minimizing the risk of thread migration and ensuring a more stable lifting effect. This refinement allows practitioners to achieve precise and natural-looking results, particularly in areas with complex anatomical structures such as the midface and jawline.

The evolution of thread thickness and flexibility has also expanded the range of applications. PDO threads are now available in varying diameters and lengths, catering to different facial regions and patient needs. Thicker threads with larger barbs are ideal for lifting heavy, sagging tissues in the lower face, while thinner threads with finer barbs are suitable for delicate areas such as the periorbital region. This variety enables practitioners to customize procedures based on individual anatomical and aesthetic requirements.

Additionally, PDO threads have been engineered to stimulate collagen production more effectively. As the threads dissolve, they induce a controlled inflammatory response, promoting fibroblast activity and the synthesis of new collagen fibers. This process not only enhances the immediate lifting effect but also improves skin elasticity, texture, and volume over time. Patients benefit from a dual effect: instant tissue repositioning and gradual skin rejuvenation that persists even after the threads have dissolved.

Another significant advancement is the integration of PDO threads with other minimally invasive techniques, such as fillers and neuromodulators. PDO threads can complement these treatments by addressing structural sagging, while fillers restore volume and neuromodulators smooth dynamic wrinkles. This combined approach maximizes the overall aesthetic outcome, providing a more harmonious and rejuvenated appearance.

The safety profile of PDO threads has also improved due to innovations in their design and insertion techniques. Modern PDO threads feature smoother surfaces and biocompatible coatings, reducing the risk of tissue trauma, inflammation, and complications such as dimpling or extrusion. The availability of blunt-tipped cannulas has further minimized procedural risks, allowing for safer insertion in high-risk areas with significant vascularity.

The comparative analysis of U-shaped and I-shaped threads reveals significant differences in their mechanical properties and clinical applications. While U-shaped threads provide stronger initial lifting effects through their suspension mechanism in the temporal area, their requirement for skin penetration at exit points presents inherent risks that must be carefully considered [14,19].

The evolution of I-shaped threads demonstrates the industry’s response to the limitations of early thread lifting techniques. These threads, while potentially providing more subtle results, offer advantages in terms of reduced tissue trauma and patient comfort. The elimination of exit points significantly reduces the risk of complications such as bleeding and visible irregularities.

Clinical outcomes suggest that the success of thread lifting procedures depends more on appropriate patient selection and customized treatment planning than on the exclusive use of any single thread type. The trend toward combining different thread types reflects a more nuanced understanding of facial aging and the need for multifaceted approaches to rejuvenation [19].

Contemporary practice indicates that optimal results are achieved through a balanced approach that considers both the mechanical properties of different thread types and the individual patient’s anatomical characteristics. This understanding has led to the development of more sophisticated treatment protocols that prioritize natural-looking results over maximum tissue elevation.

The demands for thread lifting vary across age groups. Patients in their 20 s and 30 s typically seek to enhance skin elasticity for a firm, clear complexion and prefer targeted improvements in specific areas rather than overall facial changes.

As patients enter their 40 s and 50 s, they experience a rapid decline in skin elasticity accompanied by the emergence of wrinkles. At this stage, patients require improvements in overall facial shape rather than specific areas, due to changes in innate bone structure and the condition of skin and soft tissues. The loss of deep fat layers that maintain facial volume, coupled with the loosening of ligamentous tissues that prevent sagging, results in a wider, more spread-out facial appearance. Additionally, from the 40s onwards, the reduction in collagen, elastic fibers, and sebum production, along with thinning skin, accelerates sagging and facial shape changes. Therefore, both the physical effects of barbed thread lifting for facial shape improvement and the histological effects of threads for improving loose skin, wrinkles, pigmentation, and melasma become necessary.

For those in their late 50s and 60s, skin sagging and wrinkles deepen further. Traditionally, face-lifting surgery was considered necessary for removing excess skin, with thread lifting deemed less effective. However, modern barbed threads can now lift deeper tissues including the SMAS and peri-SMAS tissues, not just skin and subcutaneous layers. This shift in concept, where moving the SMAS naturally repositions the attached skin and subcutaneous tissues along with peri-SMAS tissues, has led to an increased use of thread lifting for older patients, explaining its benefits and limitations (Figure 1). While thread lifting may not completely resolve severe sagging and deep wrinkles in elderly patients, it can alter facial shape, improve skin and soft tissue texture, and soften deep wrinkles, resulting in a naturally younger, softer, and gentler appearance. Moreover, by slowing the progression of aging, thread lifting can help maintain facial self-esteem and improve quality of life [5,20,21].

The authors primarily use PDO barbed threads. Until a few years ago, long U-shaped threads with bidirectional barbs were mainly used for lateral face lifting, while I-shaped threads with bidirectional barbs were used for anterior face lifting, gathering tissue towards the center of the thread.

This introduction provides a comprehensive overview of the evolving role of thread lifting across different age groups and its adaptation to meet varying patient needs and expectations. The authors’ approach to thread lifting has evolved in recent years, starting with a fundamental change in concept. Previously, the focus was on simply pulling skin and subcutaneous tissues upward. Now, the basic purpose of thread lifting is understood differently. When a patient is in a supine position with the chin slightly raised or the head lowered, loose tissues of the mid and lower face naturally move towards the firmer areas near the head and ears. The modern goal of thread lifting is to anchor these repositioned soft tissues to stable structures, ensuring they remain in place and do not descend again. This shift in concept led to a change in the type of threads used. Instead of bidirectional barbed threads that gather tissue towards the center of the thread, the authors now primarily use multidirectional barbed threads. These threads maintain the relocated tissue using a uniform distribution of force.

The authors emphasize the importance of thread insertion depth. While some practitioners prefer inserting multiple thin threads in the subcutaneous tissue for a light lifting effect, claiming this layer allows for smooth insertion, the authors challenge this view. Referencing the dual plane filler technique, the authors notes that the skin and subcutaneous tissues are firmly attached to the SMAS by vertically oriented fibrous septa, leaving little loose space for filler injection [22,23].

The smooth insertion of threads in the subcutaneous tissue is possible because, despite the strong attachment of skin to SMAS by fibrous septa, there are spaces between these “tree-trunk-like” septa through which threads can pass. The strength and density of these septa vary across facial areas, with areas of strong ligaments having denser septa and offering more resistance during thread insertion.

The authors argue that when threads inserted in the subcutaneous tissue pull the skin and subcutaneous tissues, the initial appearance of significant skin movement is misleading. The skin remains attached to the SMAS via fibrous septa. Pulling only the skin and subcutaneous tissues without moving the SMAS results in a temporary lifting effect that quickly diminishes as the skin returns to its original position, still connected to the unmoved SMAS.

Therefore, the authors suggest that a moderate lifting effect that feels like the underlying SMAS is appropriately adhering towards the head and ear areas is preferable to an excessive initial skin pull. This approach is believed to result in a more lasting effect.

A parallel between thread lifting and face lifting surgery is drawn, highlighting that in surgical face lifts, the SMAS is dissected and lifted to ensure long-lasting effects, as lifting only the skin and subcutaneous tissues does not provide sufficient longevity. Even surgically lifted skin tends to sag again quickly, so skin lifted only by threads is likely to return to its original position, attached to the SMAS, regardless of how tightly it is initially pulled. Excessive pulling of the skin may lead to additional problems.

The authors highlight the recent advancements in PDO dissolving threads, with a variety of thicknesses and barb designs now available. This progress has allowed I-shaped barbed threads to achieve similar effects and duration as the long U-shaped threads for lateral face lifting. I-shaped threads offer the advantage of being easier to insert, as they come preloaded in a cannula, eliminating the need for separate guide cannula insertion required for U-shaped threads.

With the significant improvements in PDO barbed thread quality, I-shaped threads can now be used to address various concerns beyond lifting sagging jawlines, including eyebrow drooping, eye area sagging, anterior cheek sagging, nasolabial folds, marionette lines, and neck sagging.

For skin rejuvenation rather than lifting, the authors prefers volume threads, multi-stranded twisted threads that provide support within the skin, over simple monofilaments.

When performing PDO barbed thread lifting, the author emphasizes several practical considerations. These include determining the direction of thread insertion based on the direction of tissue sagging, deciding on the entry points for the threads, choosing the appropriate thread thickness, number of threads, and insertion depth. Understanding the anatomy of the tissues that will be gripped by the threads and identifying important anatomical structures to avoid is also crucial. These considerations are deemed essential for any thread lifting procedure, regardless of the specific area being treated. This approach demonstrates a comprehensive understanding of both the technical aspects of thread lifting and the underlying facial anatomy, aiming to maximize effectiveness while minimizing risks.

Future research in thread lifting should prioritize the development of hybrid techniques, combining U-shaped and I-shaped threads with complementary modalities such as fillers and energy-based devices, to achieve multi-planar lifting and longer-lasting outcomes. Innovations in thread design, including enhanced biocompatibility, multidirectional barbs, and bioactive coatings, could improve safety, durability, and tissue regeneration. Long-term studies are needed to assess the longevity of results and minimize complications, while personalized approaches based on anatomical and cultural variations should guide thread selection and placement. Emerging technologies, such as smart threads with embedded sensors or bioactive agents, offer exciting possibilities for enhancing outcomes. Additionally, efforts to optimize cost-effectiveness and accessibility will ensure that thread lifting remains a viable and widely available treatment option in diverse healthcare settings.

## Figures and Tables

**Table 1:** Change in Superficial Musculo-Aponeurotic System thickness depending on facial region.

Progressive Thinning from the Preauricular Region to Medial Facial Region	
(1) Basic structure of preauricular region	− Skin, superficial fat layer with retinacular cutis superficialis, superficial fascia, deep fat layer with retinacular cutis profundus, deep fascia, masticatory muscle
(2) Parotid region	− Skin, superficial fat layer, SMAS, deep fat layer, parotid fascia, parotid capsule, parotid gland
(3) Cheek region	− Almost the same with basic structure of preauricular region
(4) Nasolabial fold region	− SMAS enveloping the mimetic muscles

**Table 2:** The key decision-making steps and criteria.

Step	Criteria/Action	Details
1. Evaluate Patient Anatomy	Assess the patient’s facial structure, soft tissue condition, and SMAS thickness.	Determine the degree of sagging, skin thickness, and the location of critical anatomical structures such as nerves and ligaments.
2. Determine Aesthetic Goals	Identify whether the patient desires dramatic lifting or subtle rejuvenation.	Clarify patient preferences for lifting intensity, downtime tolerance, and long-term results.
3. Select Appropriate Threads	Choose U-shaped, I-shaped, or a hybrid technique based on anatomy and goals.	− U-shaped Threads: Best for moderate to severe sagging and strong lifting needs in the lower face and jawline. − I-shaped Threads: Preferred for subtle lifting effects in areas like the midface and nasolabial folds. − Hybrid Techniques: Combine U-shaped threads for anchoring and I-shaped threads for contouring delicate areas.
4. Consider Procedure Complexity	Plan thread insertion based on facial zones and their structural needs.	Consider areas with higher risks of complications (e.g., vascular areas or thin skin) and adjust thread type or insertion depth to ensure safety.
5. Post-Procedure Recommendations	Outline follow-up care and management strategies tailored to the procedure performed.	Address common complications such as bruising or dimpling, provide recovery guidance, and schedule timely follow-ups to monitor results.
